# Supplementary material for: ZNF516 suppresses EGFR by targeting the CtBP/LSD1/CoREST complex to chromatin
Source: Nat Commun. 2017 Sep 25;8:691. doi: 10.1038/s41467-017-00702-5 (PMC5612949; doi:10.1038/s41467-017-00702-5)
Supplement: Supplementary file 1 — Supplementary Information [file 41467_2017_702_MOESM1_ESM.pdf]

Description of Supplementary Files

File Name: Supplementary Information
Description: Supplementary Figures and Supplementary Table

File Name: Supplementary Data 1
Description: Mass spectrometry analysis of ZNF516-associated proteins. Whole cellular extracts from HEK293T cells stably expressing FLAG-ZNF516 were immunopurified with anti-FLAG affinity column and eluted with FLAG peptides. The eluates were resolved by SDS-PAGE and silver-stained. The protein bands were retrieved and analyzed by mass spectrometry. Information on identified proteins and peptide fragments is shown.

File Name: Supplementary Data 2
Description: Cancer-related Gene Ontologies of ZNF516 & CtBP1 Bound Genes. A total of 425 genes targeted by ZNF516 and CtBP1 were identified, and then classified into various cellular biological processes using the Database for Annotation, Visualization and Integrated Discovery (DAVID, https://david.ncifcrf.gov/). Information on biological process ontologies, gene symbols, and other parameters is shown.

File Name: Peer Review File

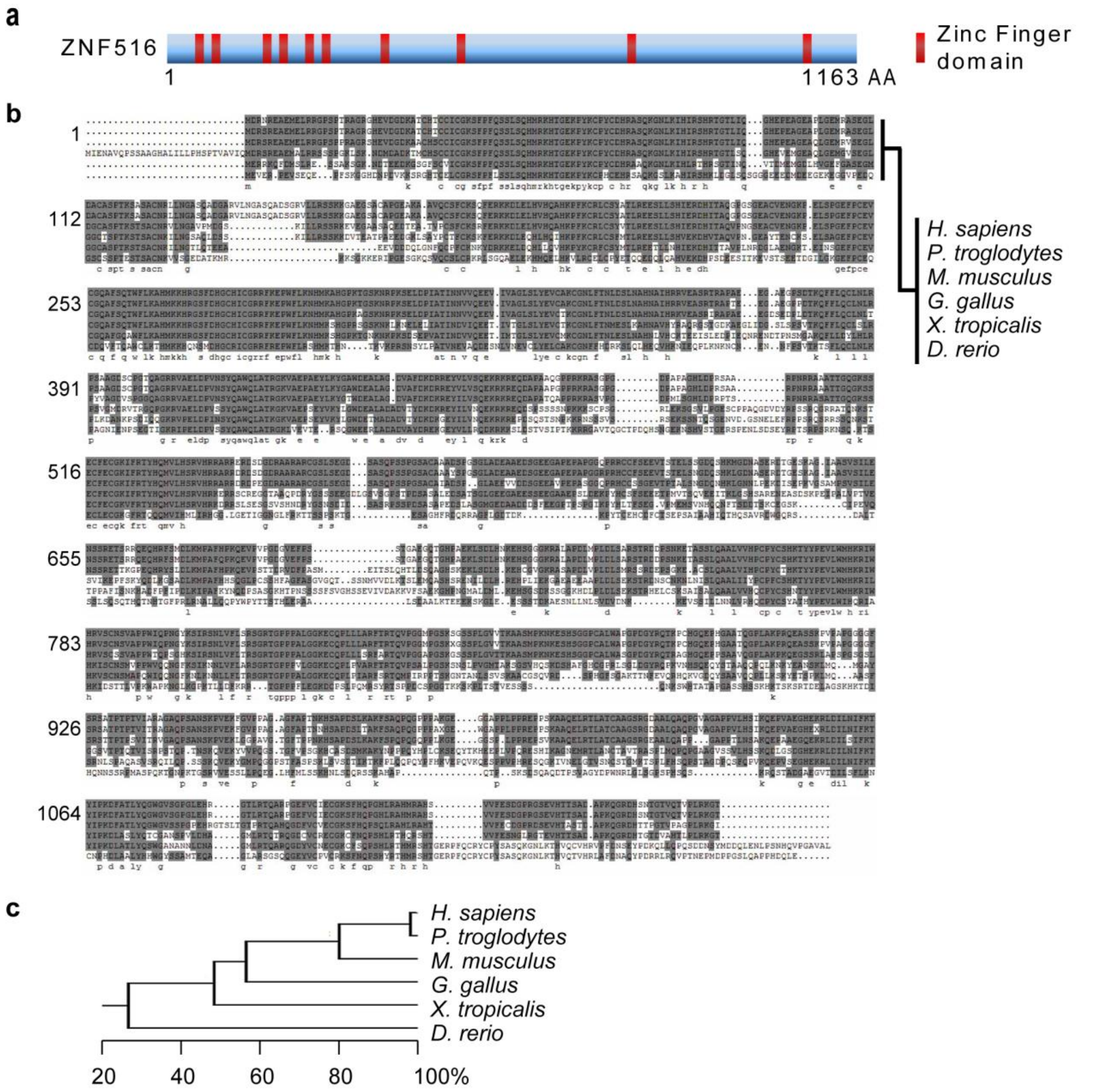

Supplementary Figure 1. ZNF516 Is an Evolutionarily Well-conserved Gene. (a) A schematic representation of the structure of ZNF516. The 10 conserved domains of zinc finger are shown. (b) Amino-acid sequence alignment of ZNF516 from different species. Shaded residues represent conserved region. (c) Phylogenetic analysis of evolutionary relationships among homologs of ZNF516 proteins from different species.

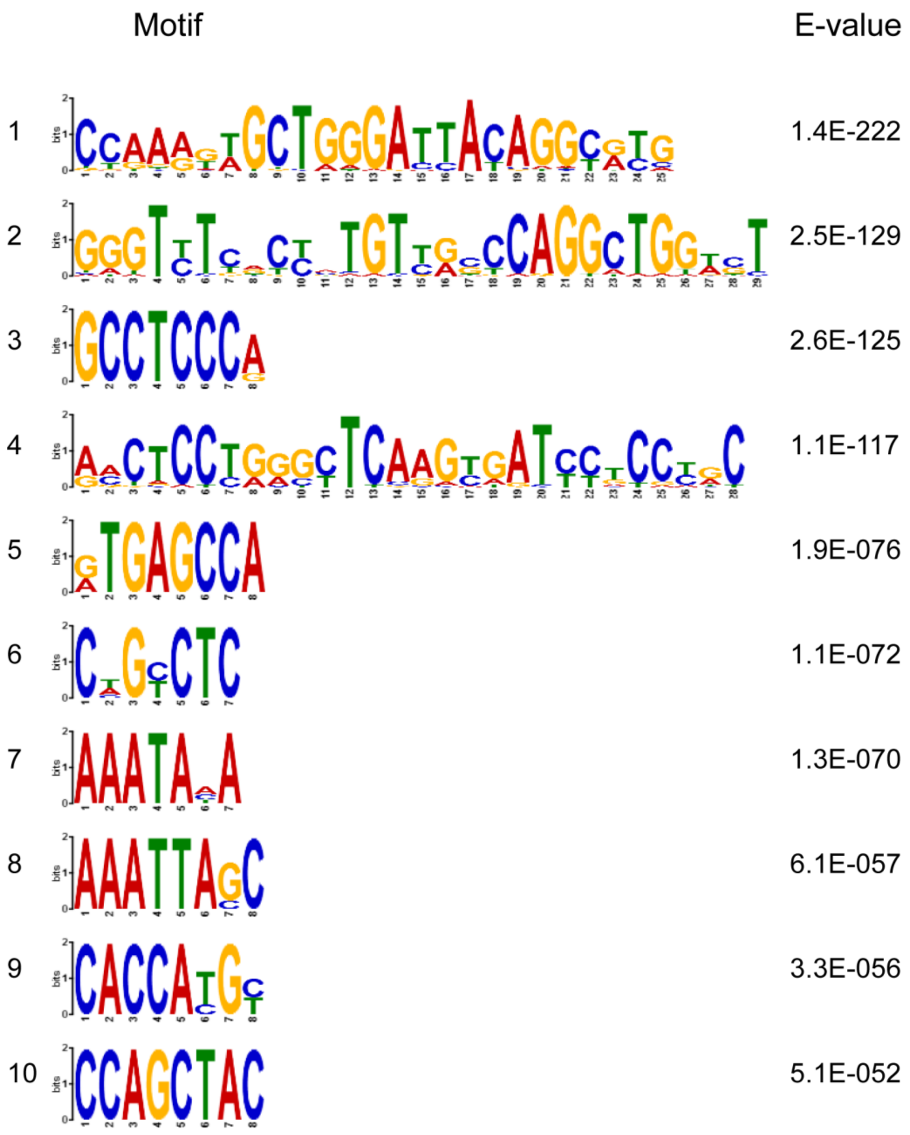

Supplementary Figure 2. The Top 10 Consensus Binding Motifs Enriched at ZNF516 Peaks via MEME-ChIP Analysis. Significance is ranked according to E-values as labeled. The E-value of a motif is based on its log likelihood ratio, width, number of sites, the background letter frequencies, and the size of the training set.

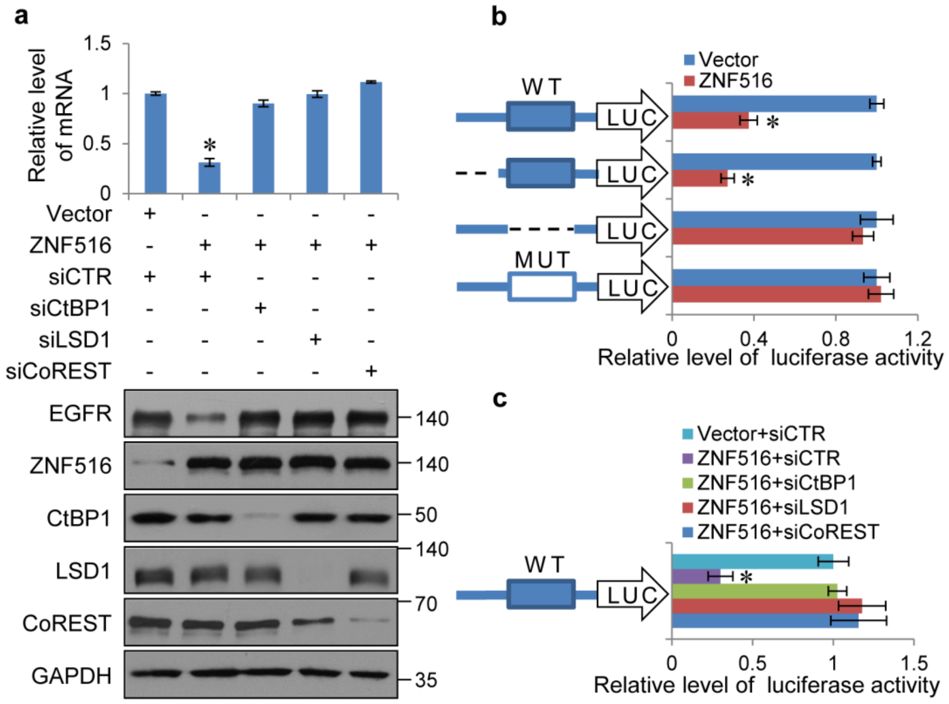

Supplementary Figure 3. Transcription Repression of EGFR by the ZNF516-CtBP/LSD1/CoREST Complex in MDA-MB-231 Cells. (a) MDA-MB-231 cells were transfected with siRNAs of control, CtBP1, LSD1, or CoREST together with empty vector or ZNF516 expression constructs. The mRNA and protein level of EGFR was measured by real-time RT-PCR and Western blotting, respectively. Error bars represent mean ± S.D. for three independent experiments. *p*-values were determined by Student's *t*-test. (*) $p < 0.05$. (b) MDA-MB-231 cells were co-transfected with *EGFR*-Luc wild-type, or mutants and expression construct for ZNF516. Forty-eight hours after the transfection, luciferase activity was measured. Relative luciferase activity was calculated as firefly luciferase activity divided by renilla luciferase activity and shown relative to the control. Each bar represents the mean ± S.D. for triplicate experiments. *p*-values were determined by Student's *t*-test. (*) $p < 0.05$. (c) MDA-MB-231 cells were transfected with control, CtBP1, LSD1 or CoREST siRNAs and/or ZNF516 expression constructs together with *EGFR*-Luc construct. Forty-eight hours after the transfection, luciferase activity was measured. Relative luciferase activity was calculated as firefly luciferase activity divided by renilla luciferase activity and shown relative to the control. Each bar represents the mean ± S.D. for triplicate experiments. *p*-values were determined by Student's *t*-test. (*) $p < 0.05$.

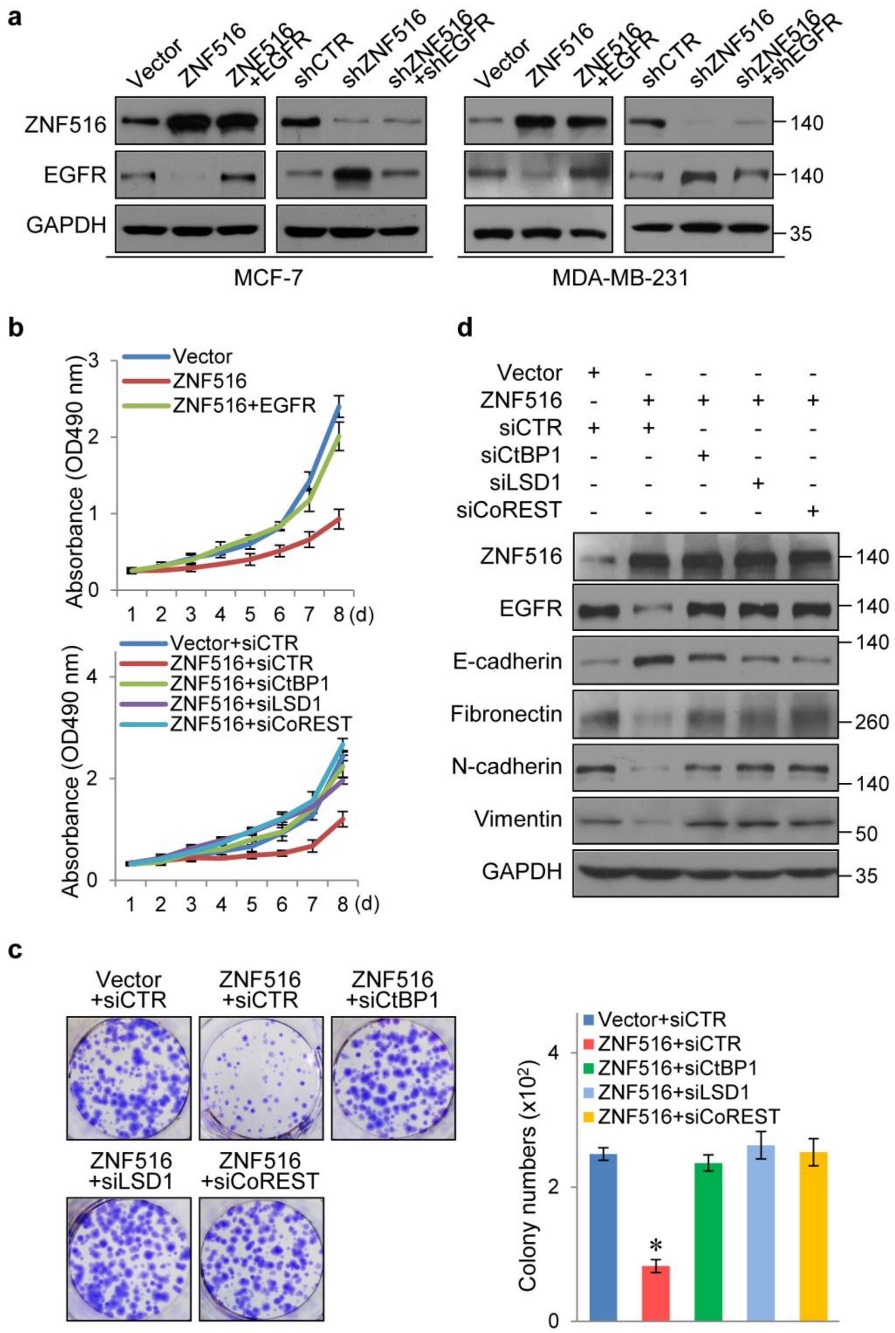

Supplementary Figure 4. The ZNF516-CtBP/LSD1/CoREST Complex Inhibits the Proliferation and Invasion of MDA-MB-231 Breast Cancer Cells. (a) MCF-7 or MDA-MB-231 cells were infected with lentiviruses carrying FLAG-tagged ZNF516 or/and EGFR, or with lentiviruses carrying ZNF516 shRNA or/and EGFR shRNA, and treated with puromycin and/or neomycin. Single cells were then manually isolated and maintained for small colony formation. Small colony was picked up for continuous culture and the levels of ZNF516 and EGFR were confirmed by WB analysis. For the re-expressing and re-silencing EGFR experiments, the level of EGFR expression was controlled

by creating stable clones of cells that were expressing different levels of EGFR, and the clones with EGFR levels close to original EGFR level were chosen for phenotype experiments. (b) MDA-MB-231 cells were stably infected with lentiviruses carrying FLAG-tagged ZNF516 or/and EGFR (upper), or transfected with the control, CtBP1, LSD1, or CoREST siRNAs together with expression constructs for ZNF516 (lower). Cells were split into 96-well plates and then harvested at indicated day. The growth curves of the cells were measured with MTS assay. Each point represents the mean ± S.D. for three independent experiments. (c) MDA-MB-231 cells transfected with the control, CtBP1, LSD1, or CoREST siRNAs together with expression constructs for ZNF516 were maintained for 14 days before staining with crystal violet and counting for colony numbers in colony formation assay. Each bar represents the mean ± S.D. for three independent experiments. p-values were determined by Student's t-test. (*) $p < 0.05$. (d) The expressions of the epithelial and mesenchymal markers were measured by Western blotting in MDA-MB-231 cells co-transfected with the siRNAs of control, CtBP1, LSD1, or CoREST and the expression constructs for ZNF516.

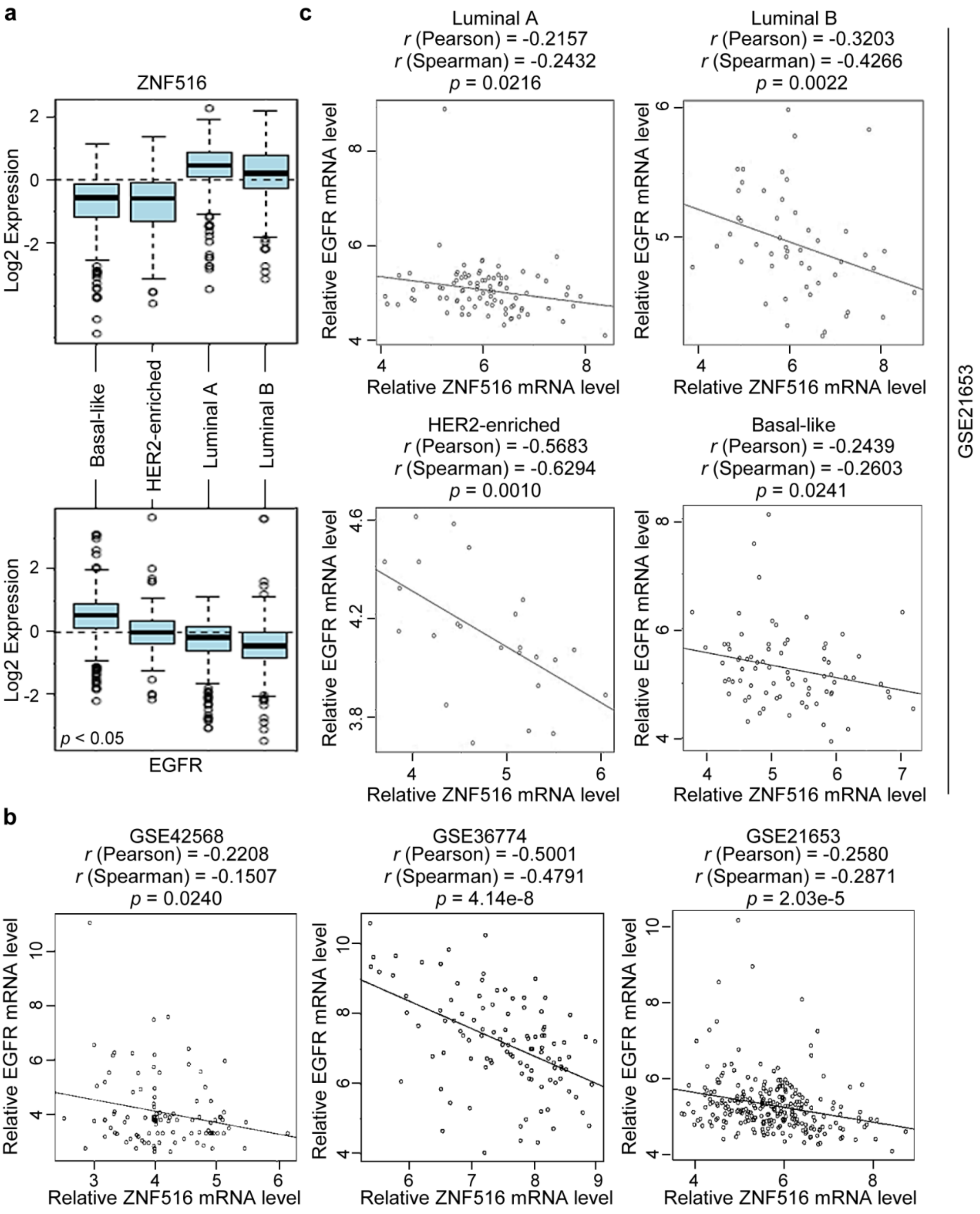

a
ZNF516
Log2 Expression
Basal-like
HER2-enriched
Luminal A
Luminal B
Log2 Expression
$p < 0.05$
EGFR
c
Luminal A
r (Pearson) = -0.2157
r (Spearman) = -0.2432
p = 0.0216
Luminal B
r (Pearson) = -0.3203
r (Spearman) = -0.4266
p = 0.0022
HER2-enriched
r (Pearson) = -0.5683
r (Spearman) = -0.6294
p = 0.0010
Basal-like
r (Pearson) = -0.2439
r (Spearman) = -0.2603
p = 0.0241
Relative EGFR mRNA level
Relative ZNF516 mRNA level
GSE21653
b
GSE42568
r (Pearson) = -0.2208
r (Spearman) = -0.1507
p = 0.0240
GSE36774
r (Pearson) = -0.5001
r (Spearman) = -0.4791
p = 4.14e-8
GSE21653
r (Pearson) = -0.2580
r (Spearman) = -0.2871
p = 2.03e-5
Relative EGFR mRNA level
Relative ZNF516 mRNA level

d

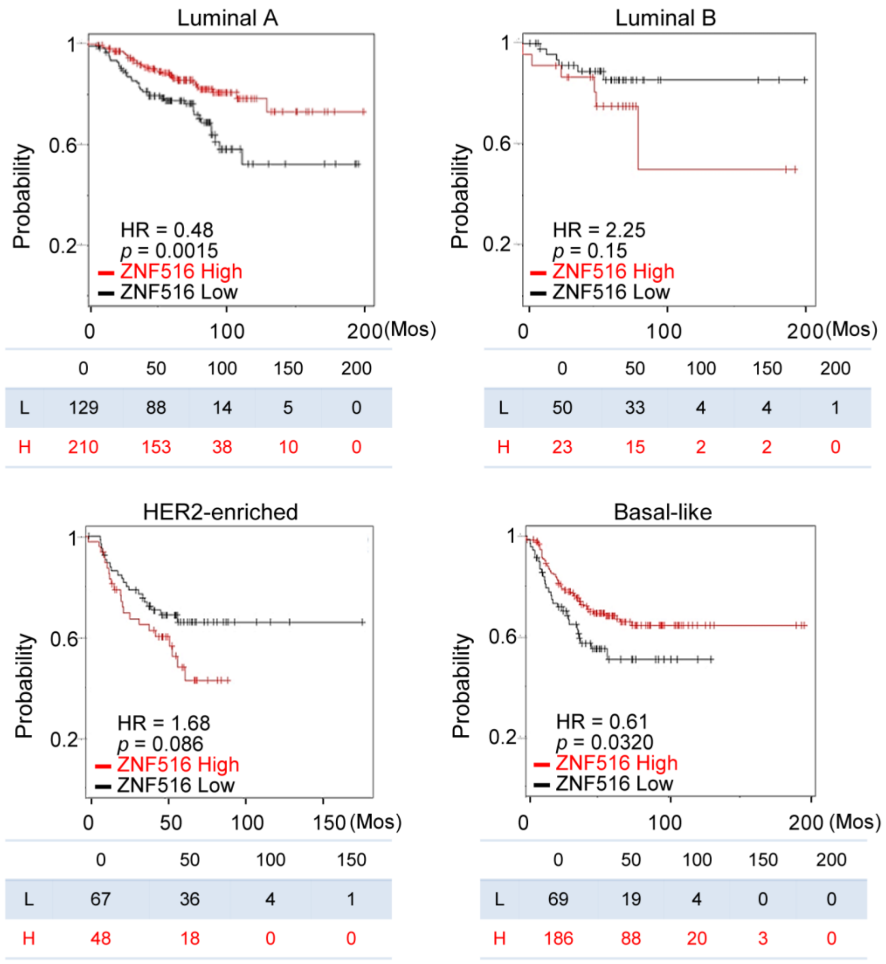

Supplementary Figure 5. The Expression Level of ZNF516 Is Negatively Correlated with that of EGFR, and Low Expression of ZNF516 Is Positively Correlated with Poor Patience Survival in Luminal A and Basal-like Breast Cancer. (a) The expression of ZNF516 and EGFR in different subtypes of breast cancer via the Gene expression-based Outcome for Breast cancer Online (GOBO) analysis (http://co.bmc.lu.se/gobo/gsa.pl). (b) Analysis of public datasets (GSE42568, GSE36774, and GSE21653) for correlation of ZNF516 and EGFR expression. The relative level of EGFR was plotted against that of ZNF516. (c) Analysis of public dataset (GSE21653) for correlation of mRNA expression between ZNF516 and EGFR in luminal A, luminal B, HER2-enriched, and basal-like breast cancer. The relative level of EGFR was plotted against that of ZNF516. (d) The association of ZNF516 expression and relapse-free survival in luminal A, luminal B, HER2-enriched, and basal-like breast via Kaplan-Meier survival analysis (http://kmplot.com/analysis/).

Supplementary Figure 6

Uncropped blots related to Figure 1a

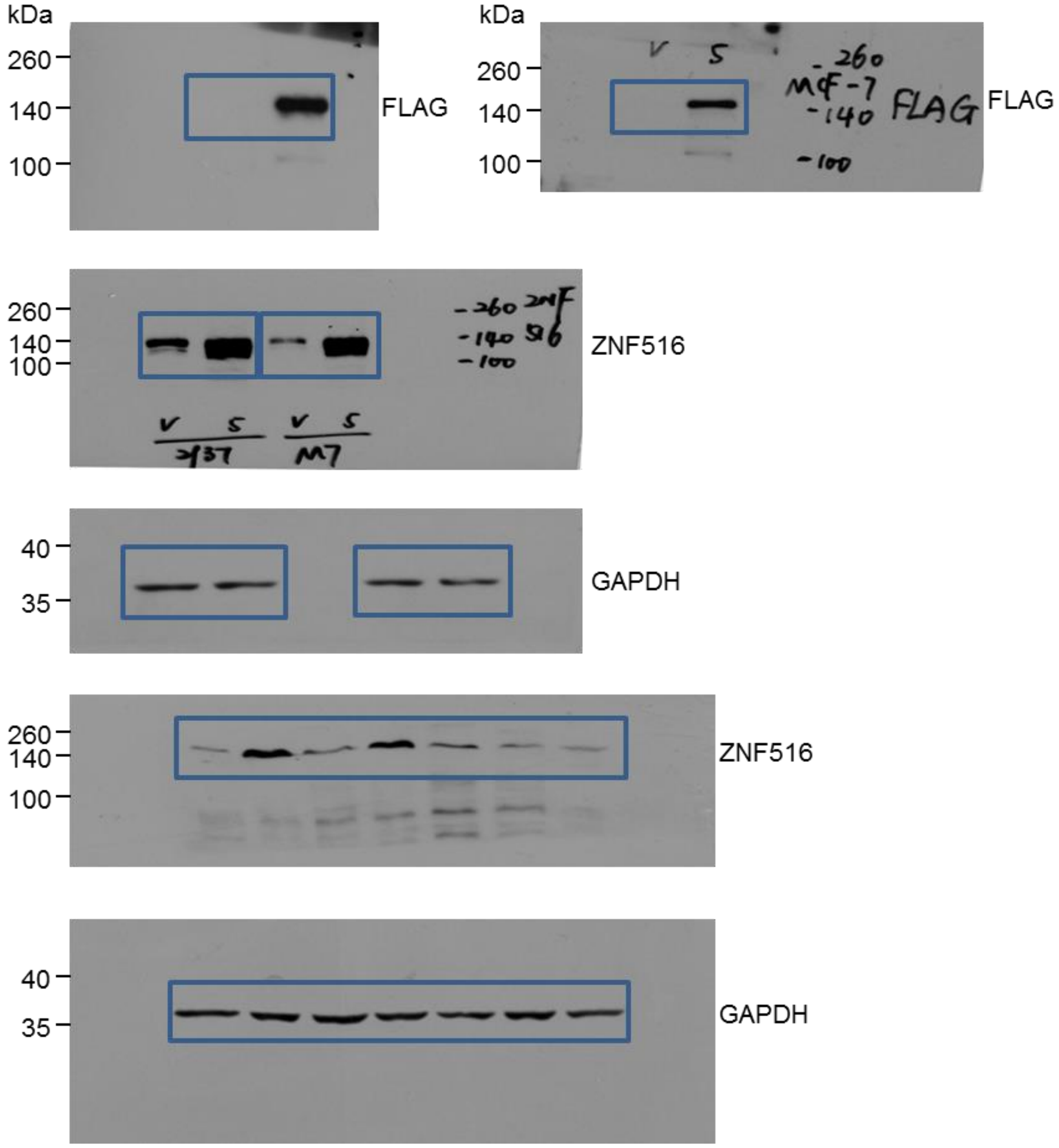

Supplementary Figure 6 (continued)

Uncropped blots related to Figure 1e

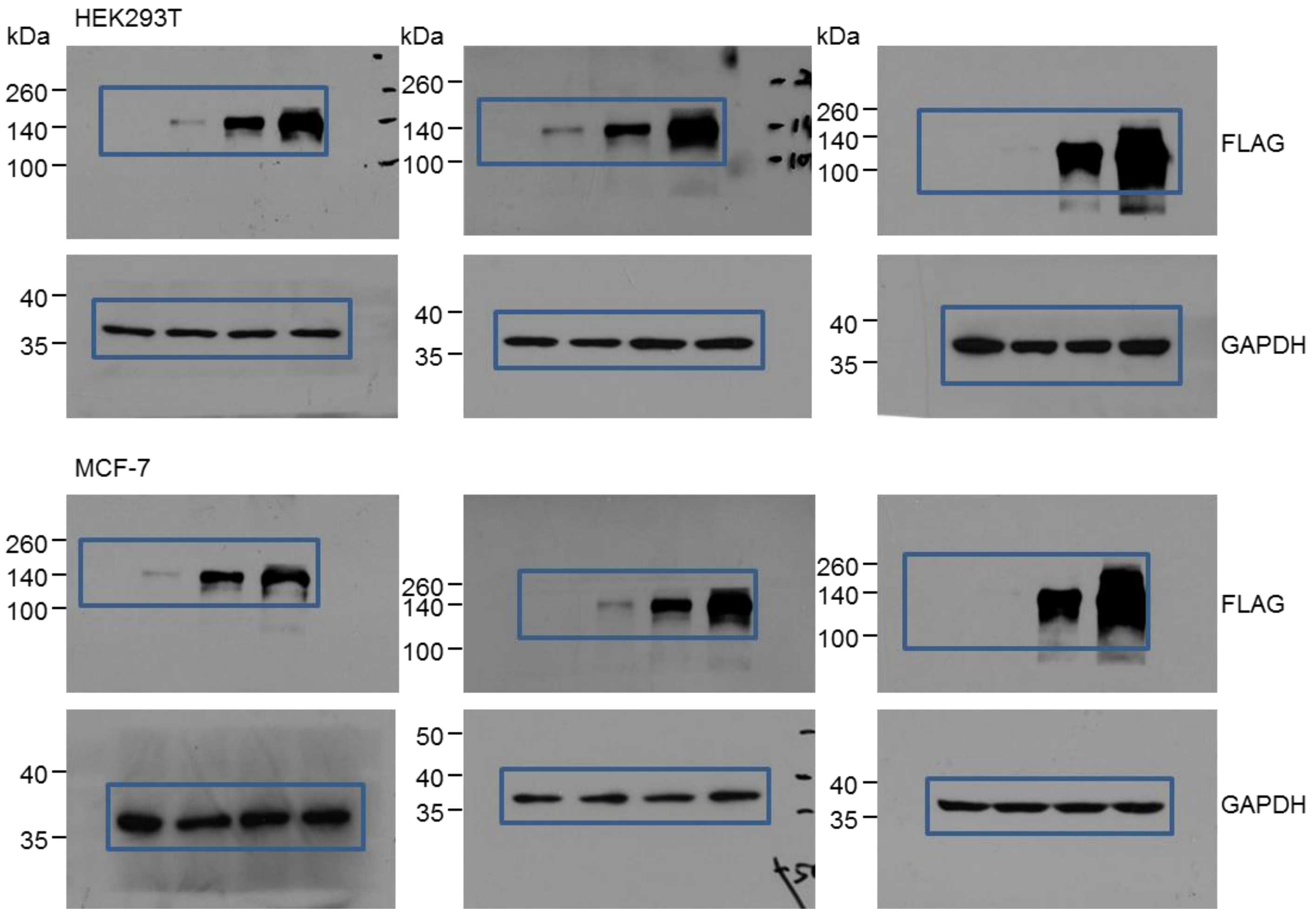

Uncropped blots related to Figure 2b

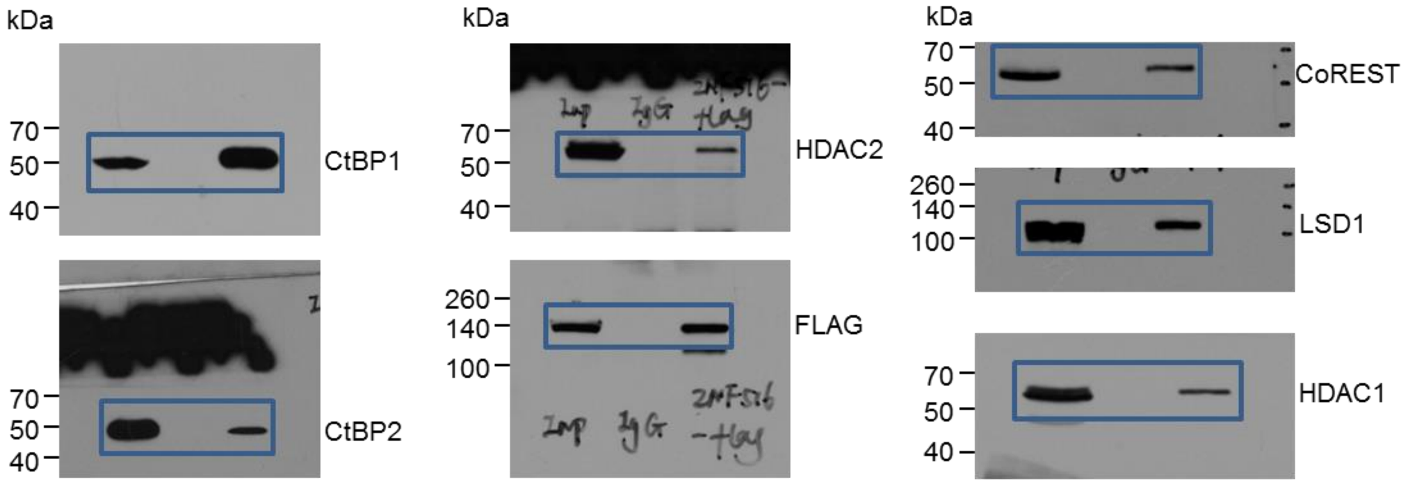

Supplementary Figure 6 (continued)

Uncropped blots related to Figure 2c

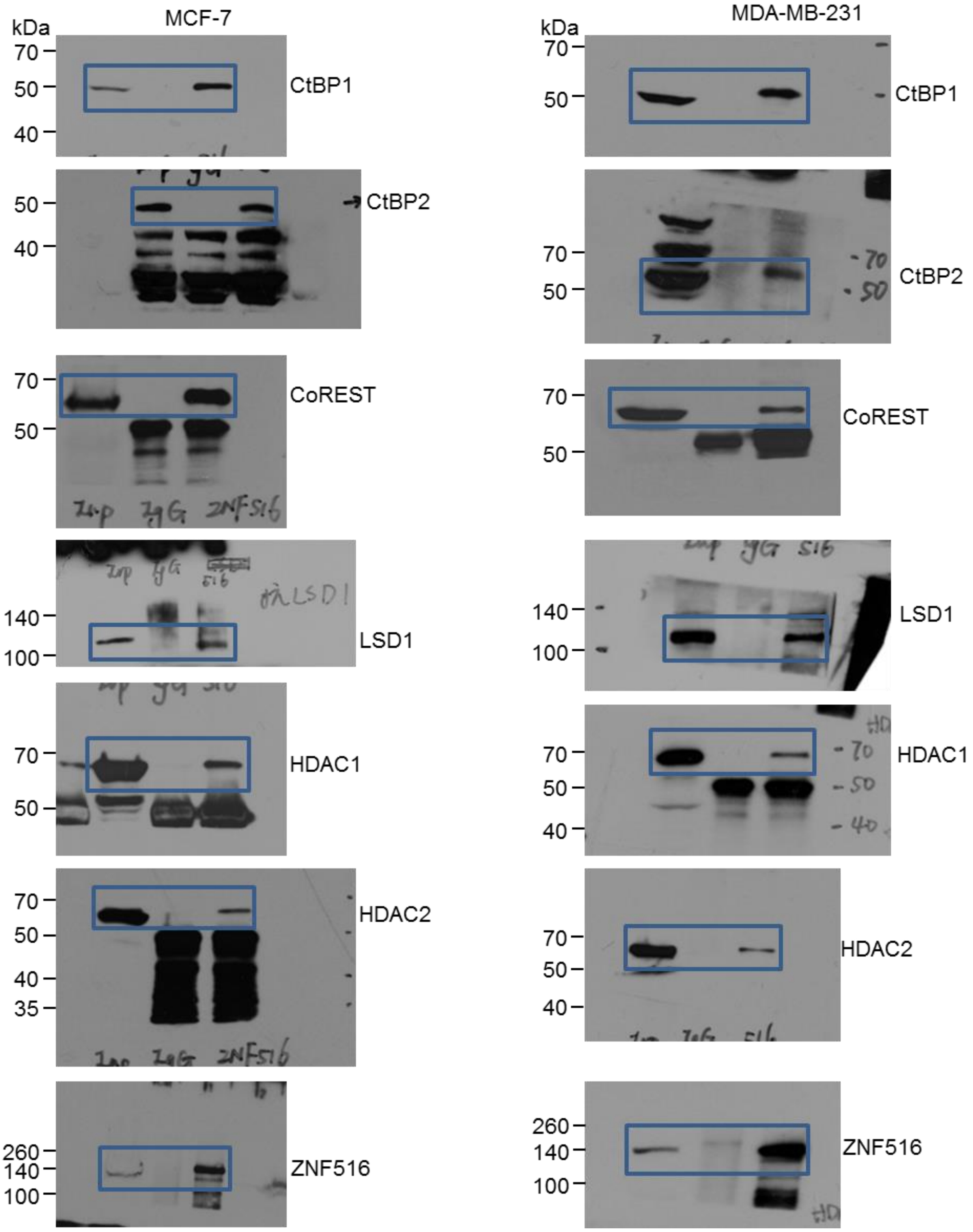

Uncropped blots related to Figure 2d

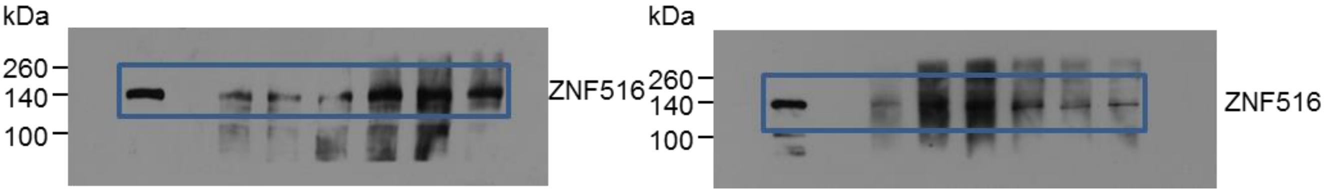

Supplementary Figure 6 (continued)

Uncropped blots related to Figure 3a

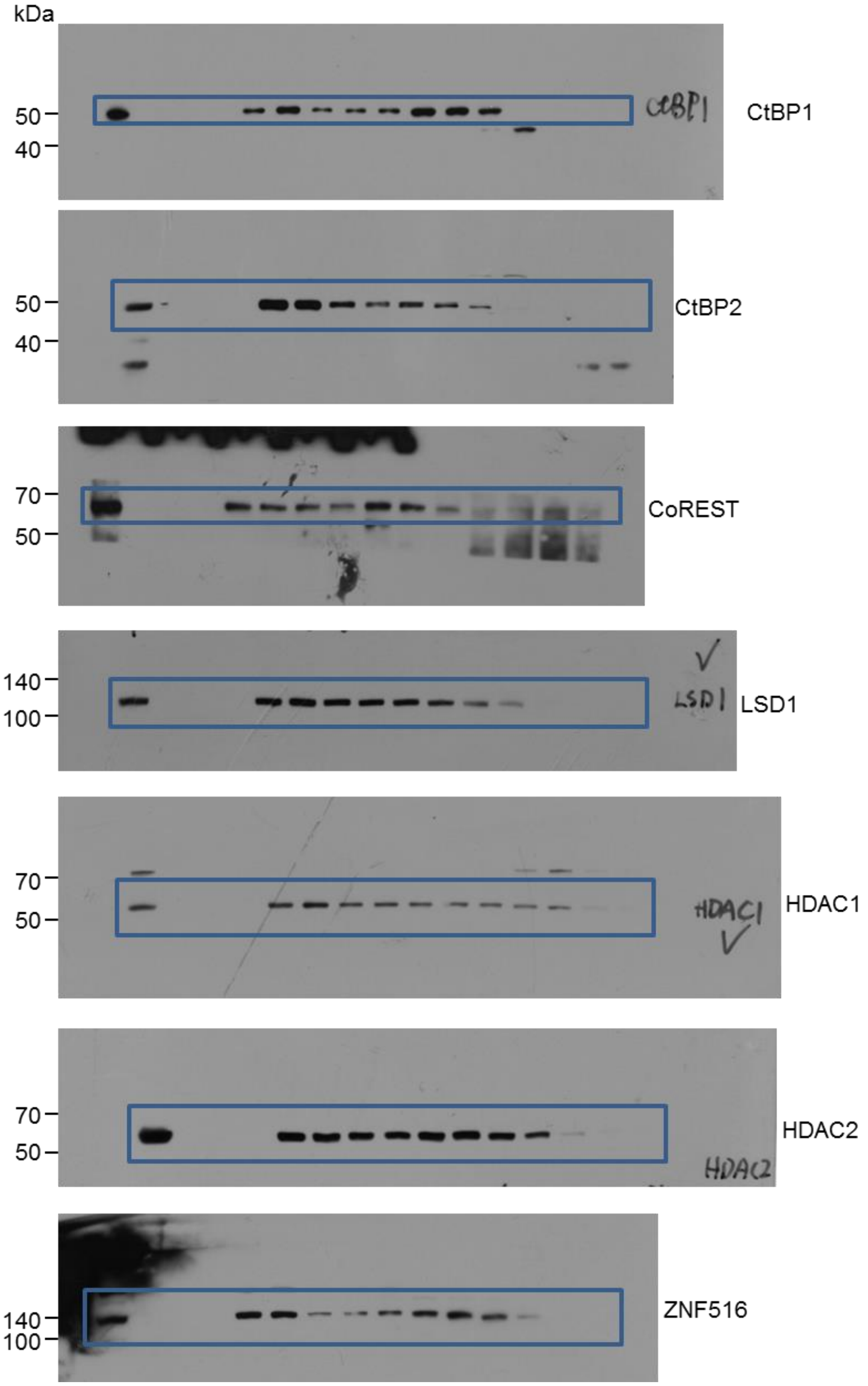

Supplementary Figure 6 (continued)

Uncropped blots related to Figure 3b

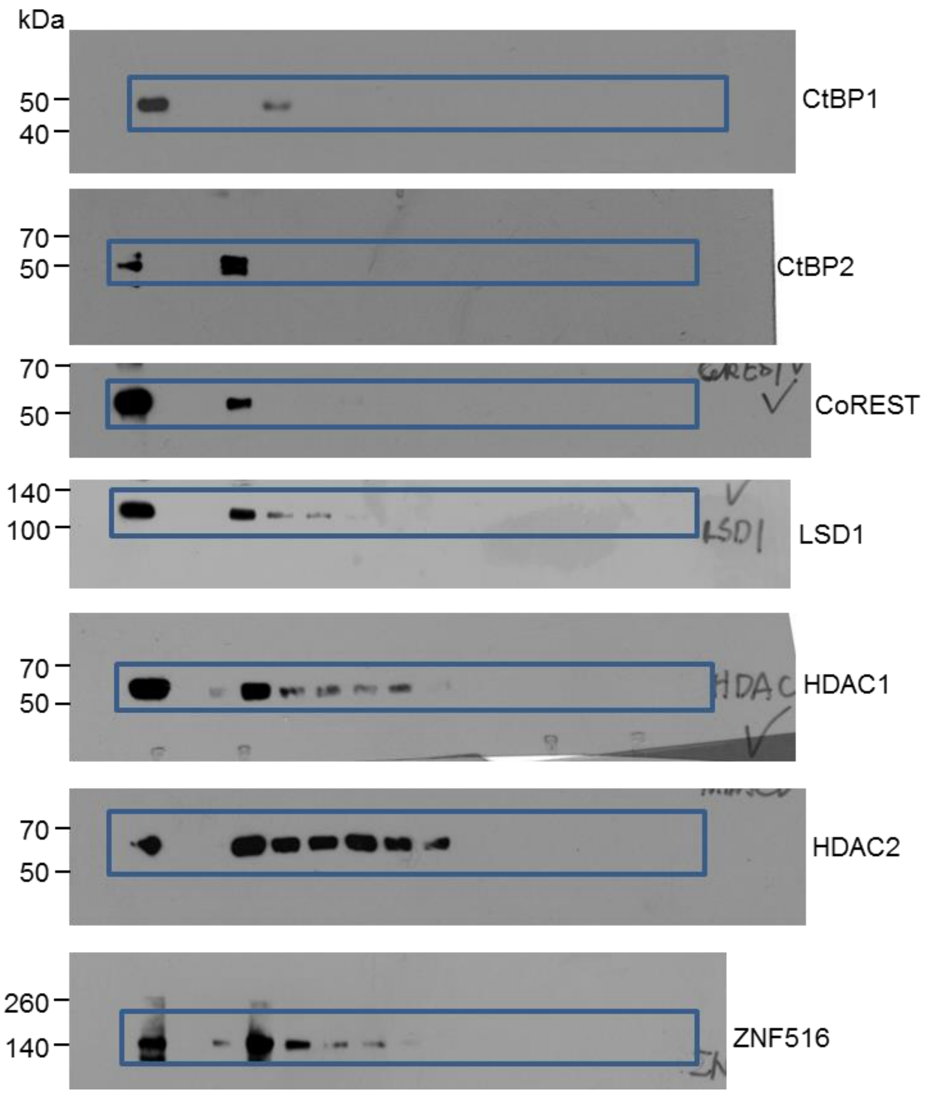

Uncropped blots related to Figure 3d

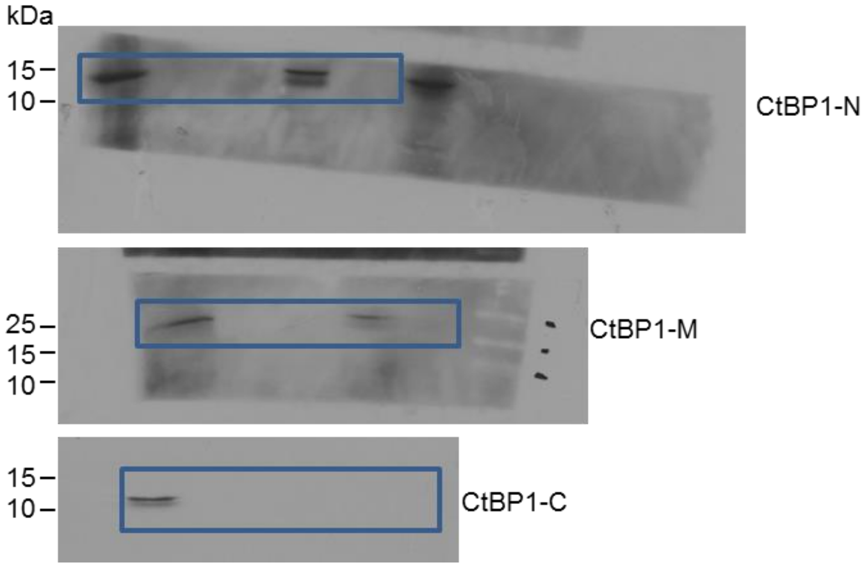

Supplementary Figure 6 (continued)

Uncropped blots related to Figure 3d

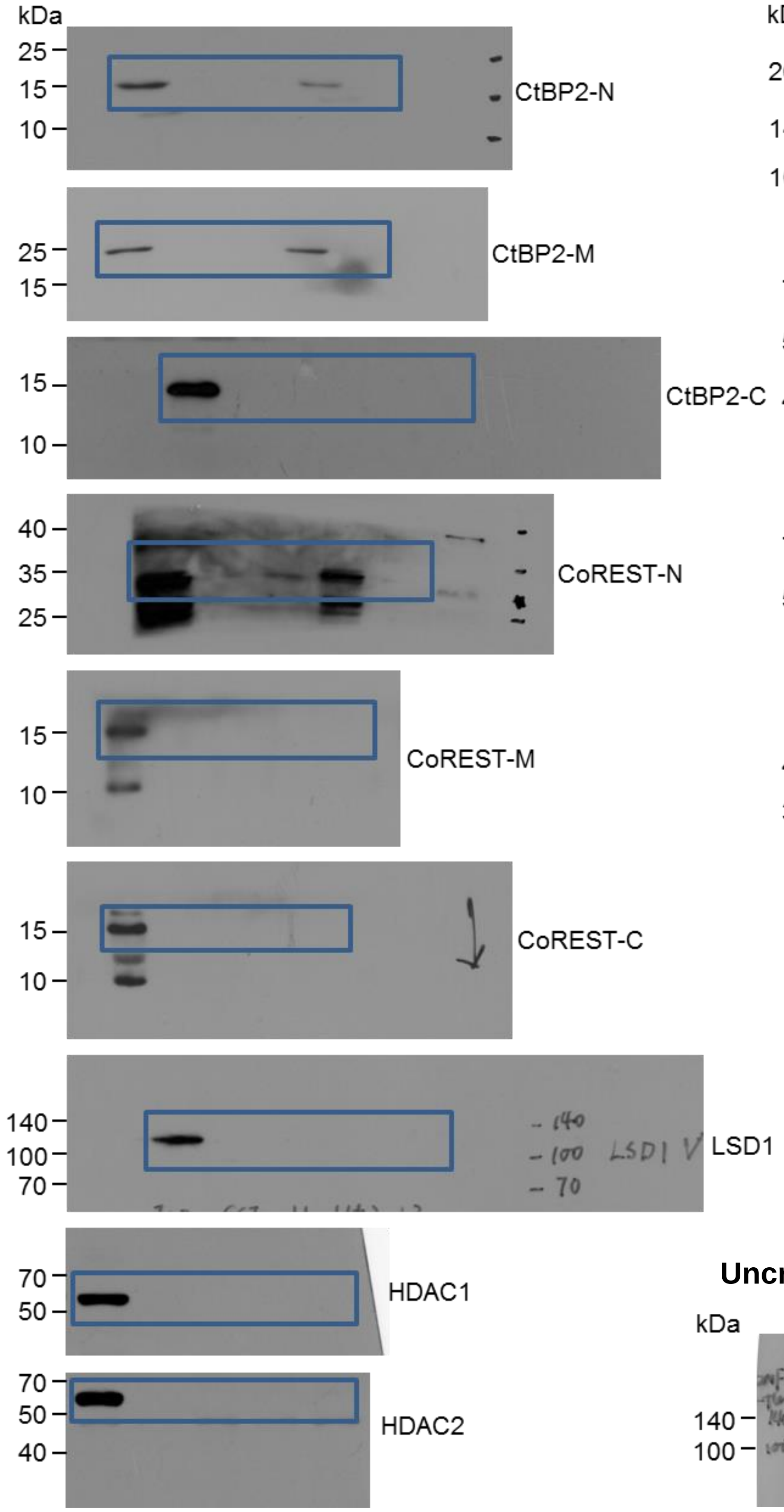

Uncropped blots related to Figure 5d

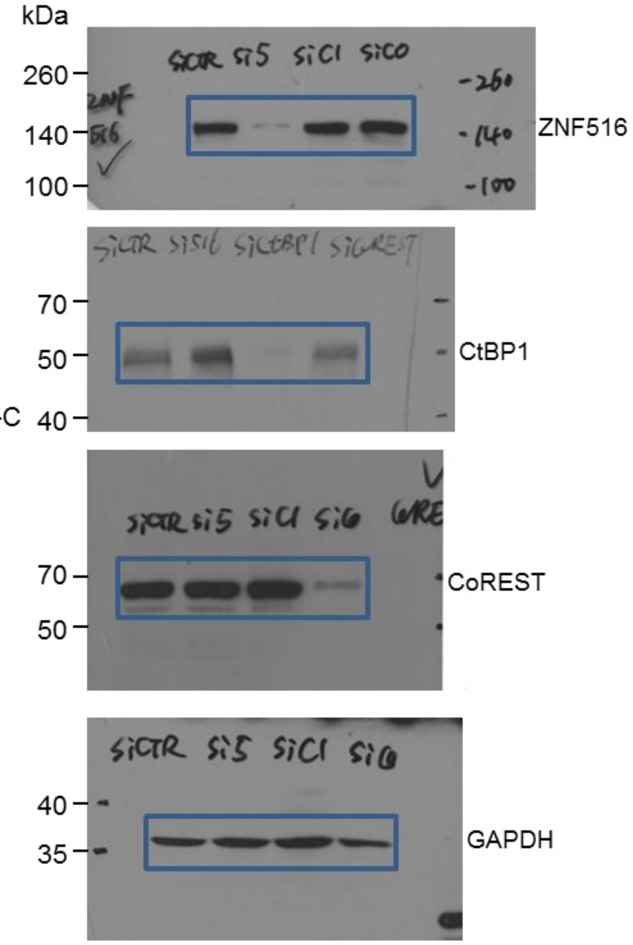

Uncropped blots related to Figure 3e

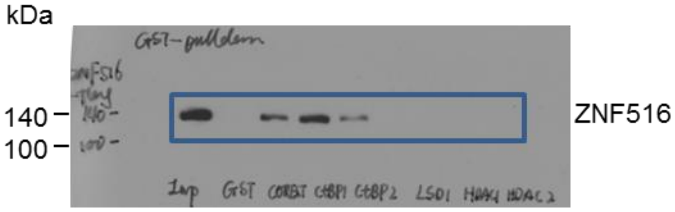

Supplementary Figure 6 (continued)

Uncropped blots related to Figure 6a

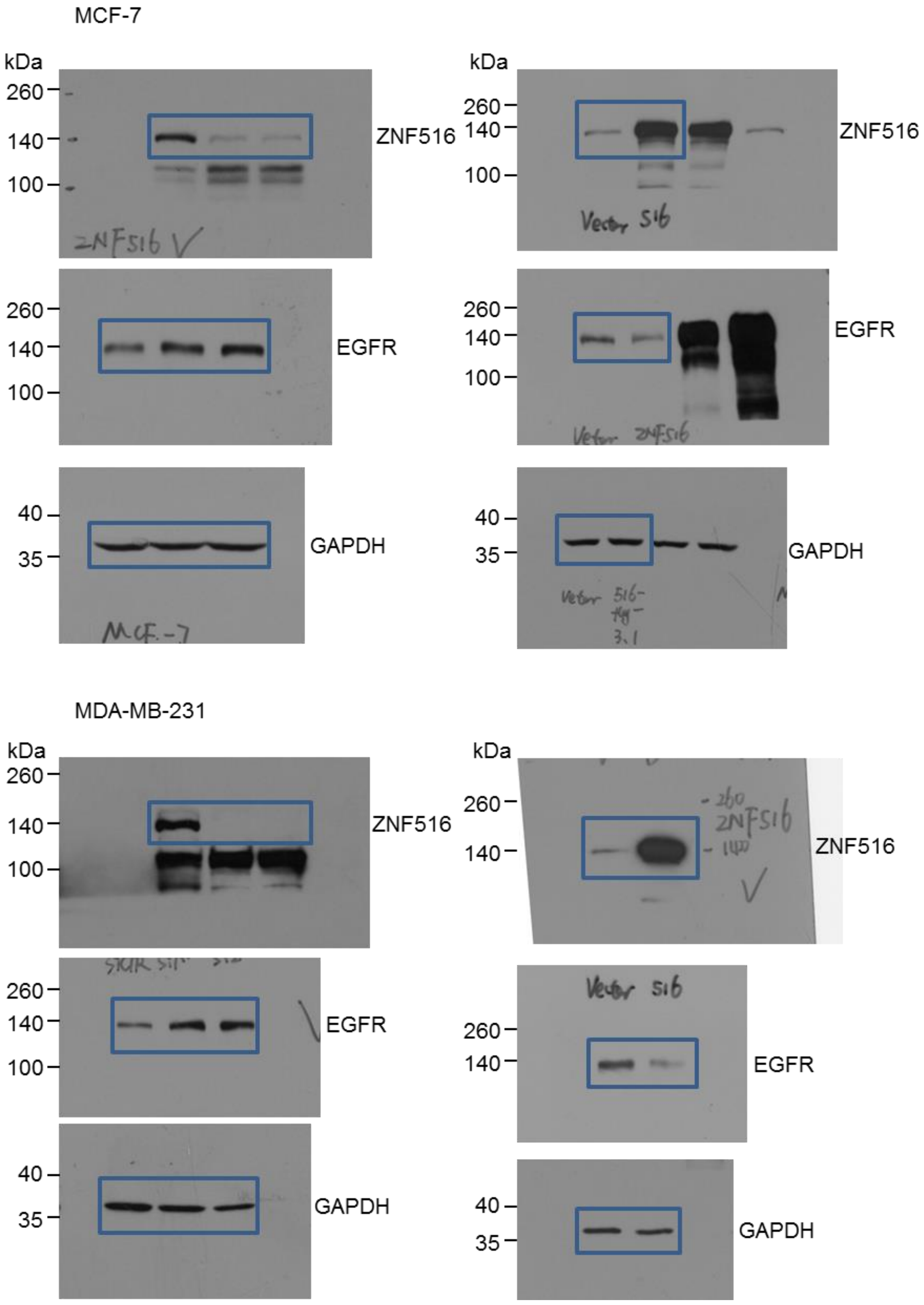

Supplementary Figure 6 (continued)

Uncropped blots related to Figure 6b

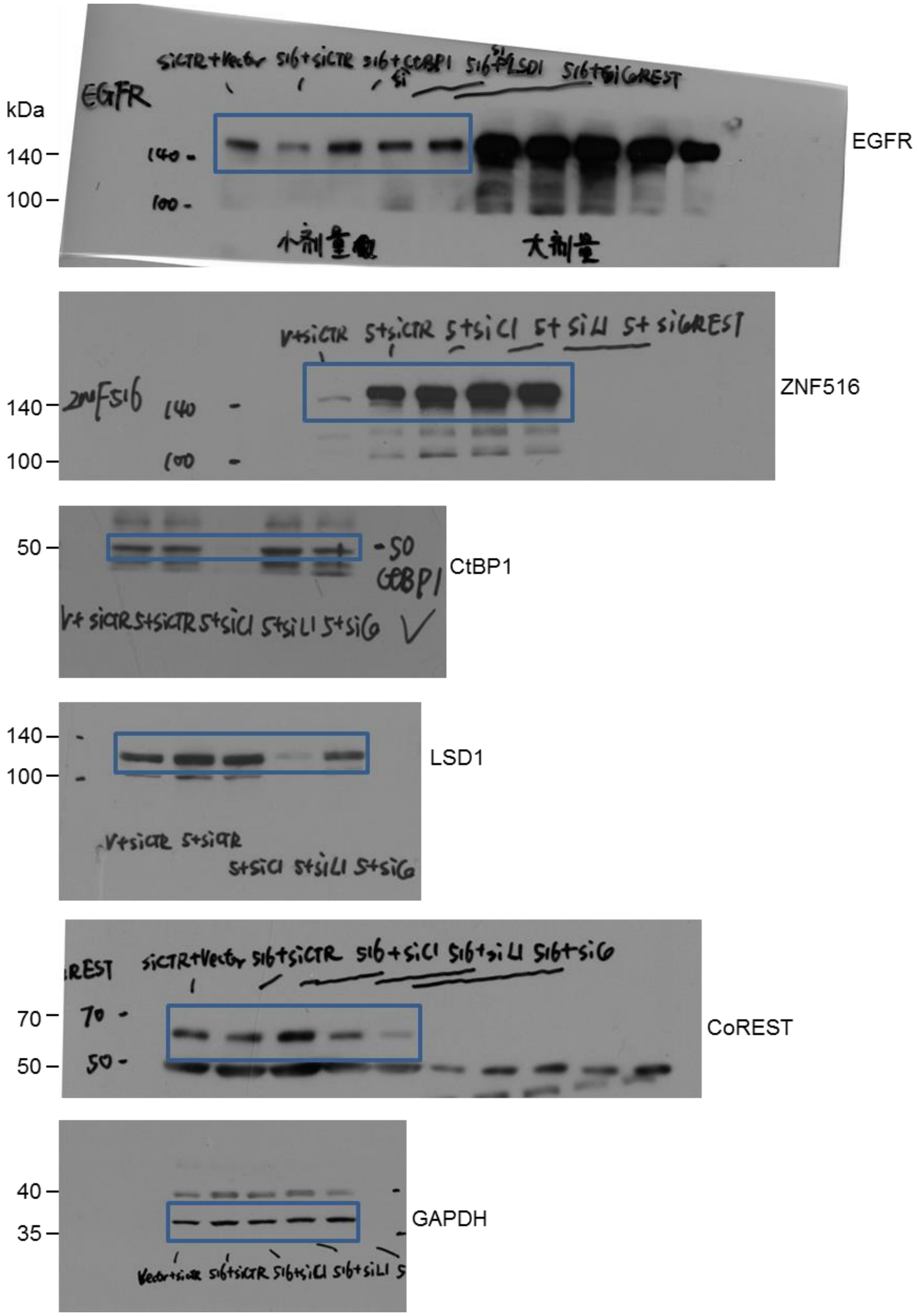

Supplementary Figure 6 (continued)

Uncropped blots related to Figure 6e

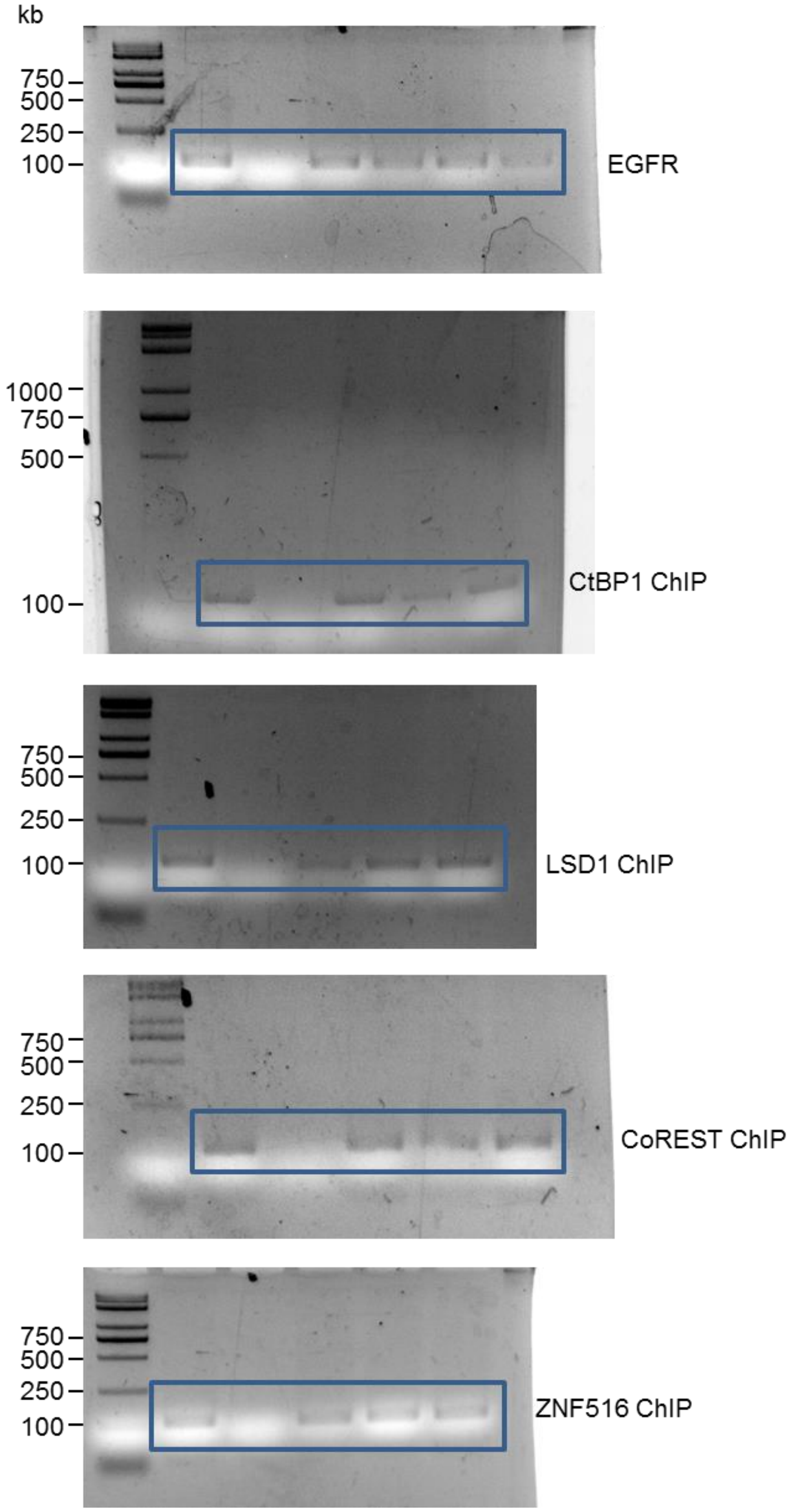

Supplementary Figure 6 (continued)

Uncropped blots related to Figure 7d

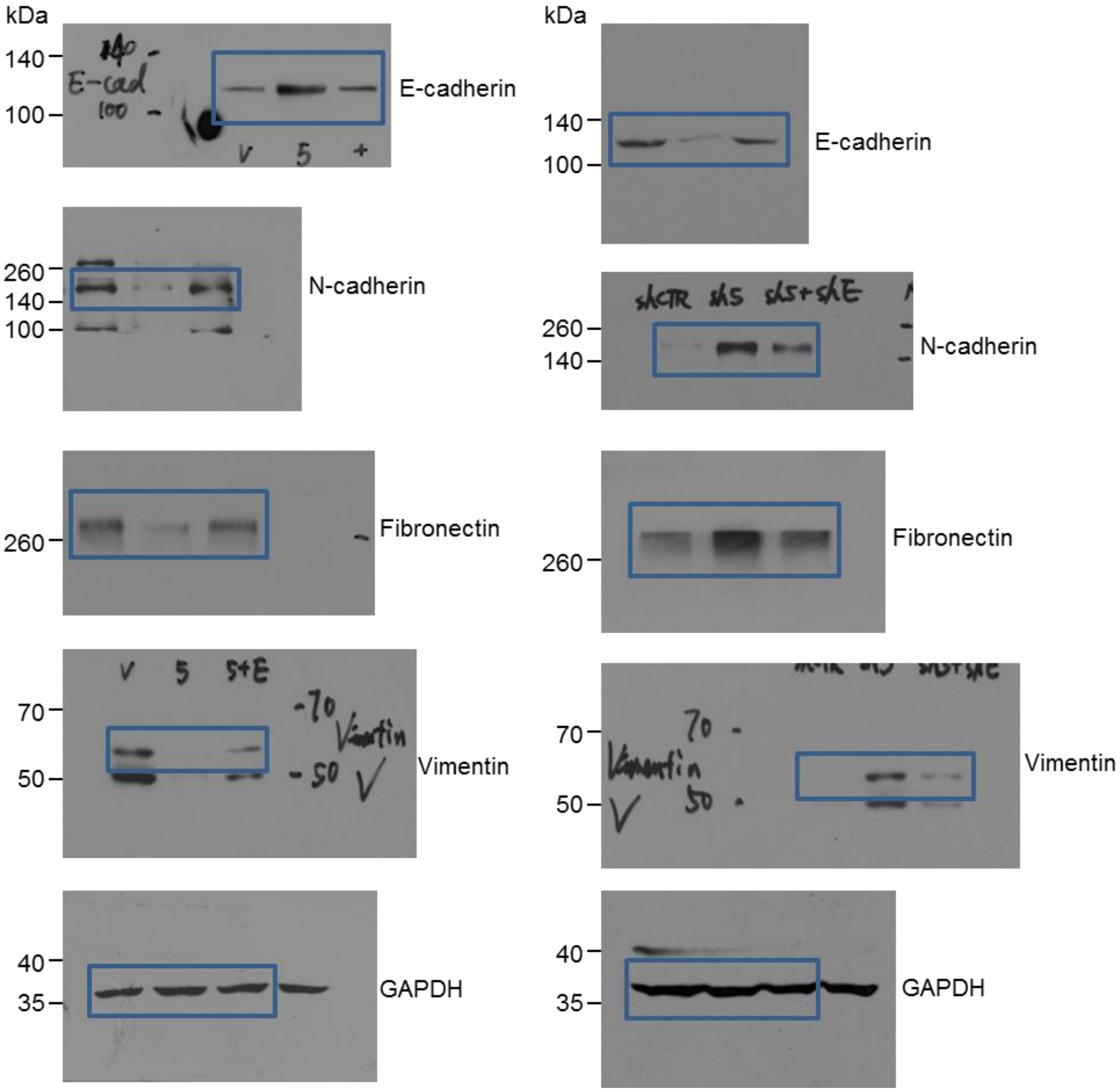

Supplementary Figure 6 (continued)

Uncropped blots related to Figure 7e

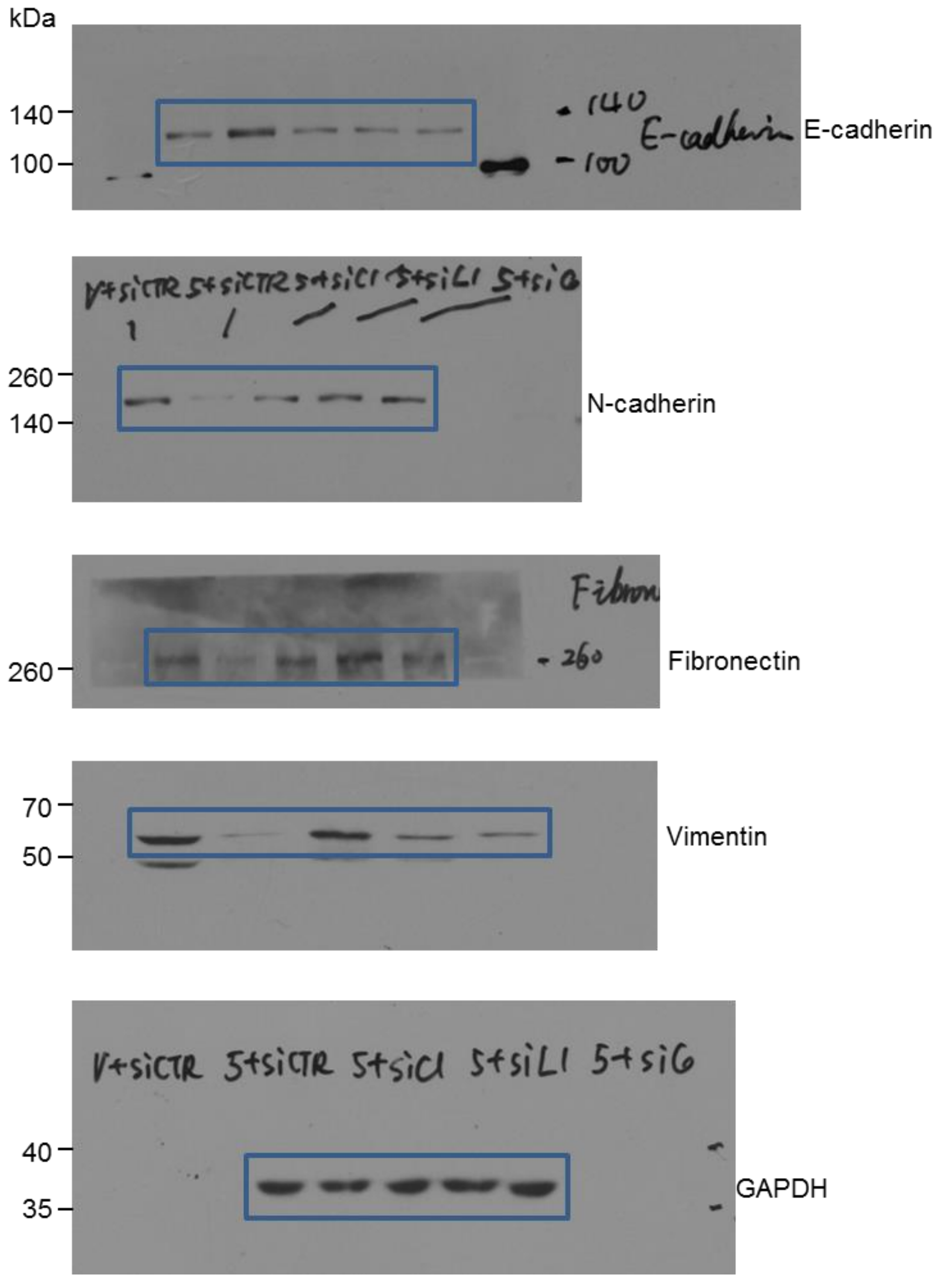

Supplementary Figure 6 (continued)

Uncropped blots related to Figure 8a

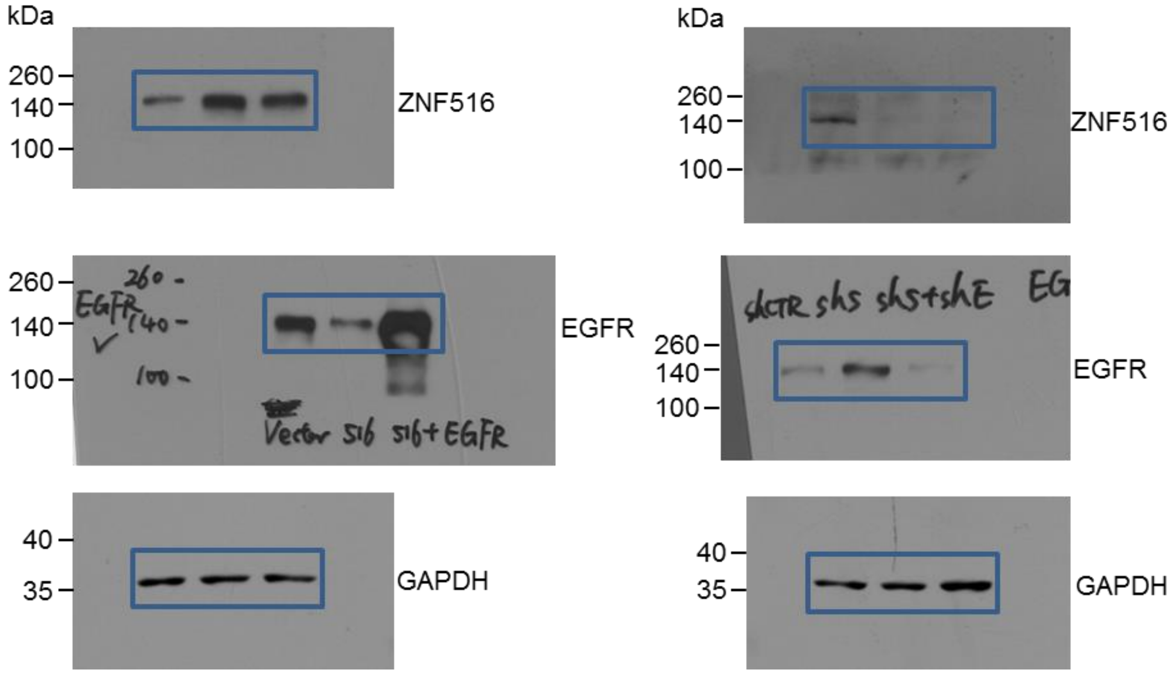

Uncropped blots related to Figure 9a

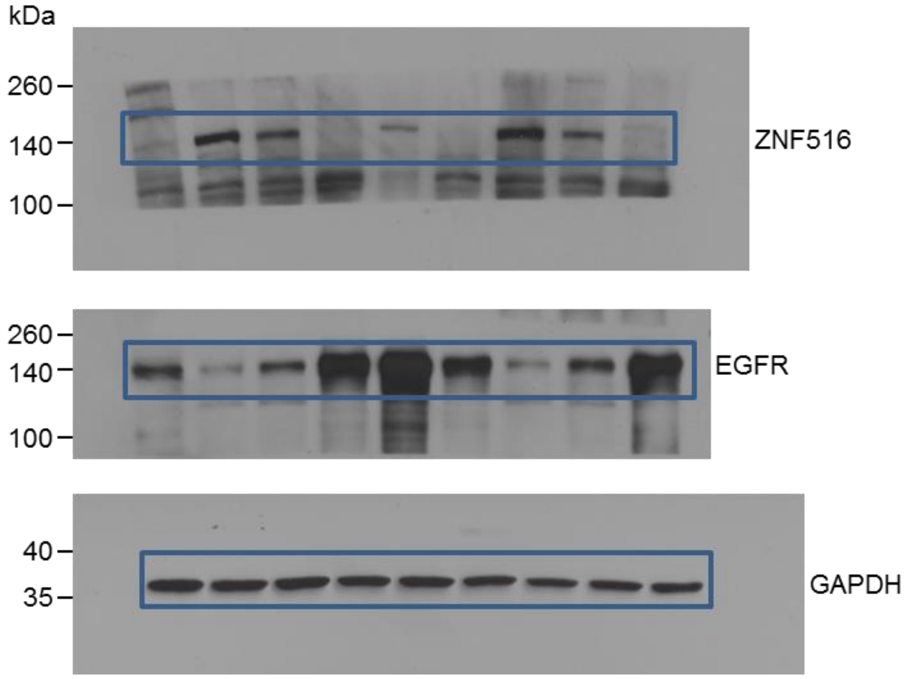

Supplementary Figure 6 (continued)

Uncropped blots related to Figure 9b

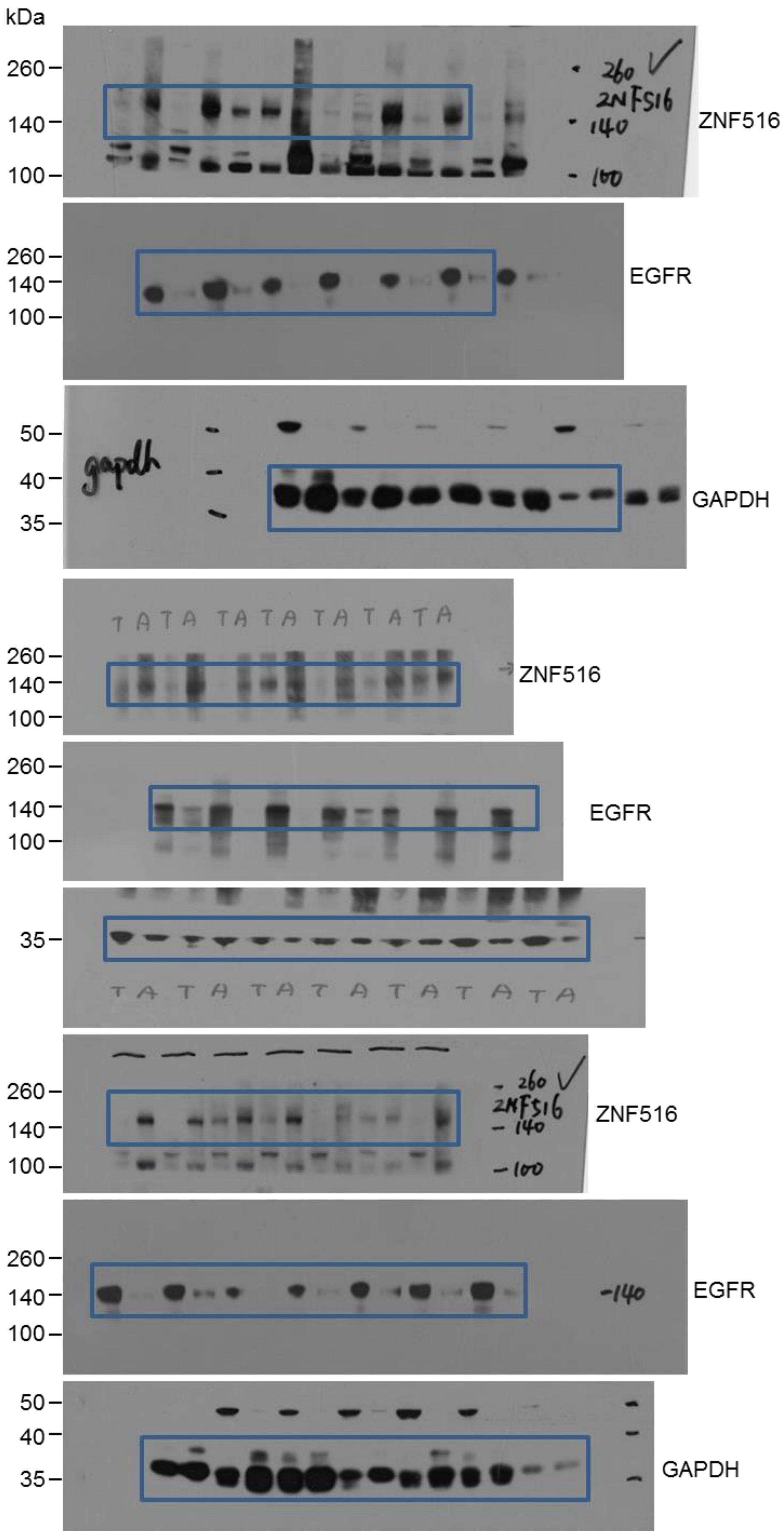

Supplementary Figure 6 (continued)

Uncropped blots related to Supplementary Figure 3

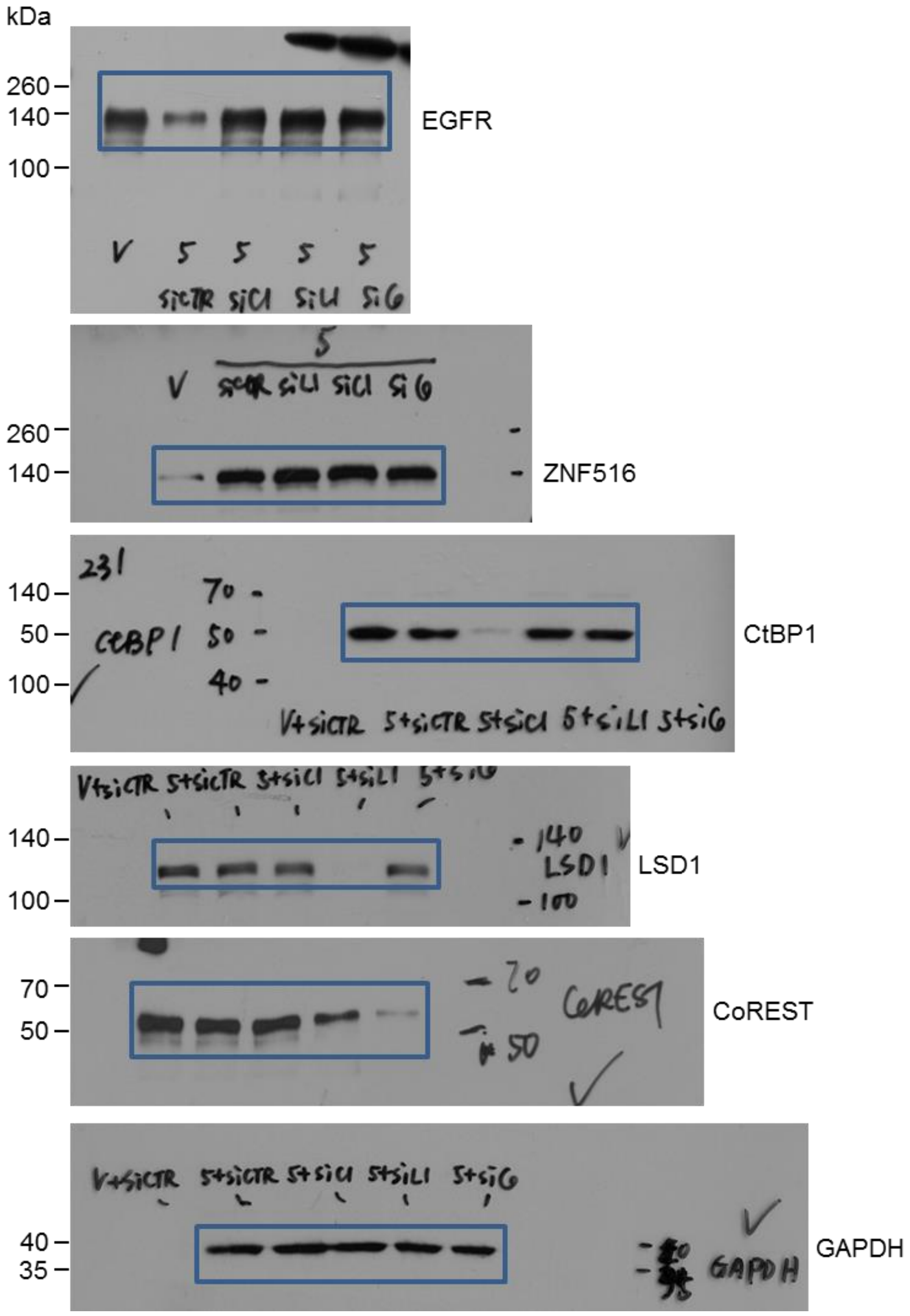

Supplementary Figure 6 (continued)

Uncropped blots related to Supplementary Figure 4a

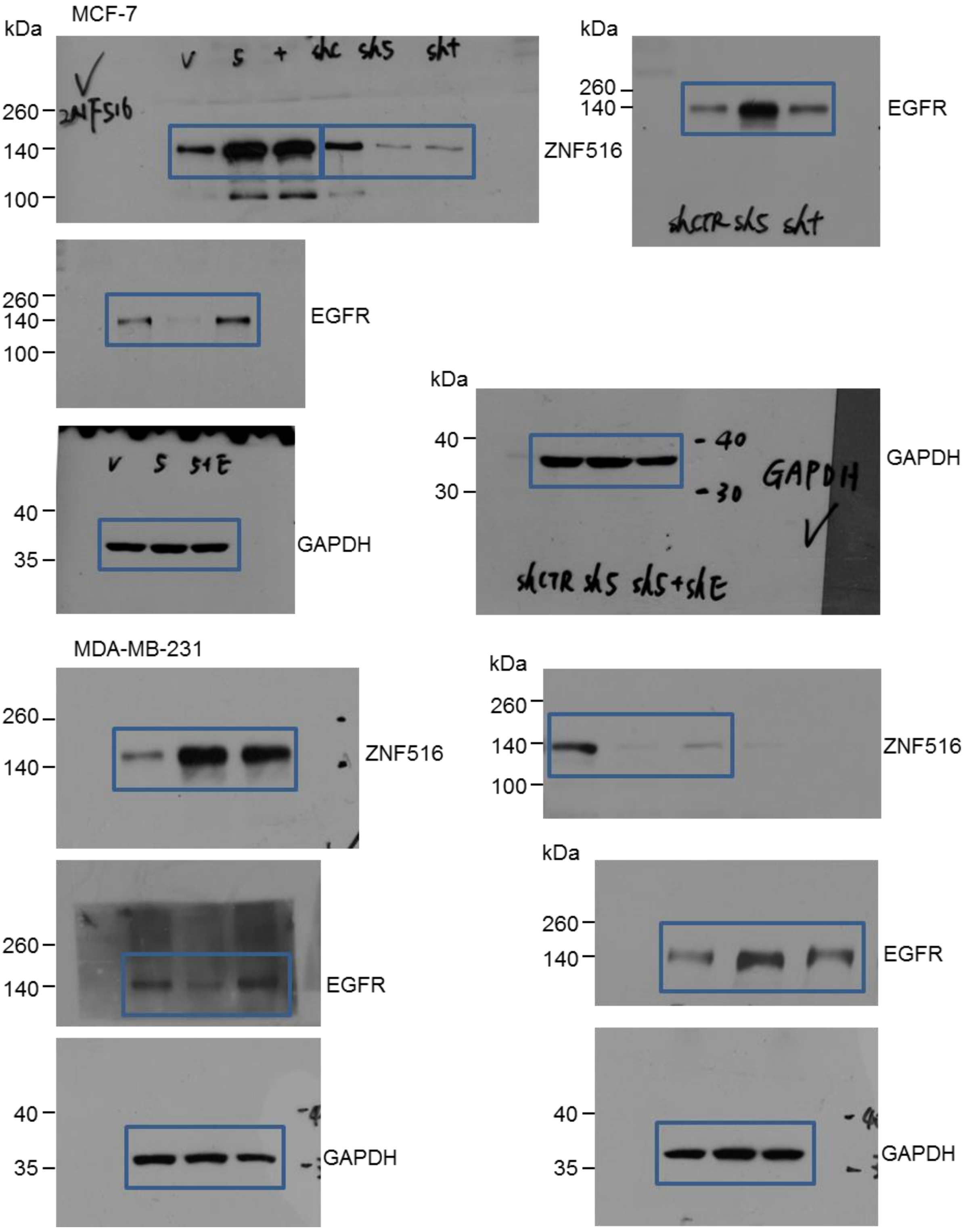

Supplementary Figure 6 (continued)

Uncropped blots related to Supplementary Figure 4d

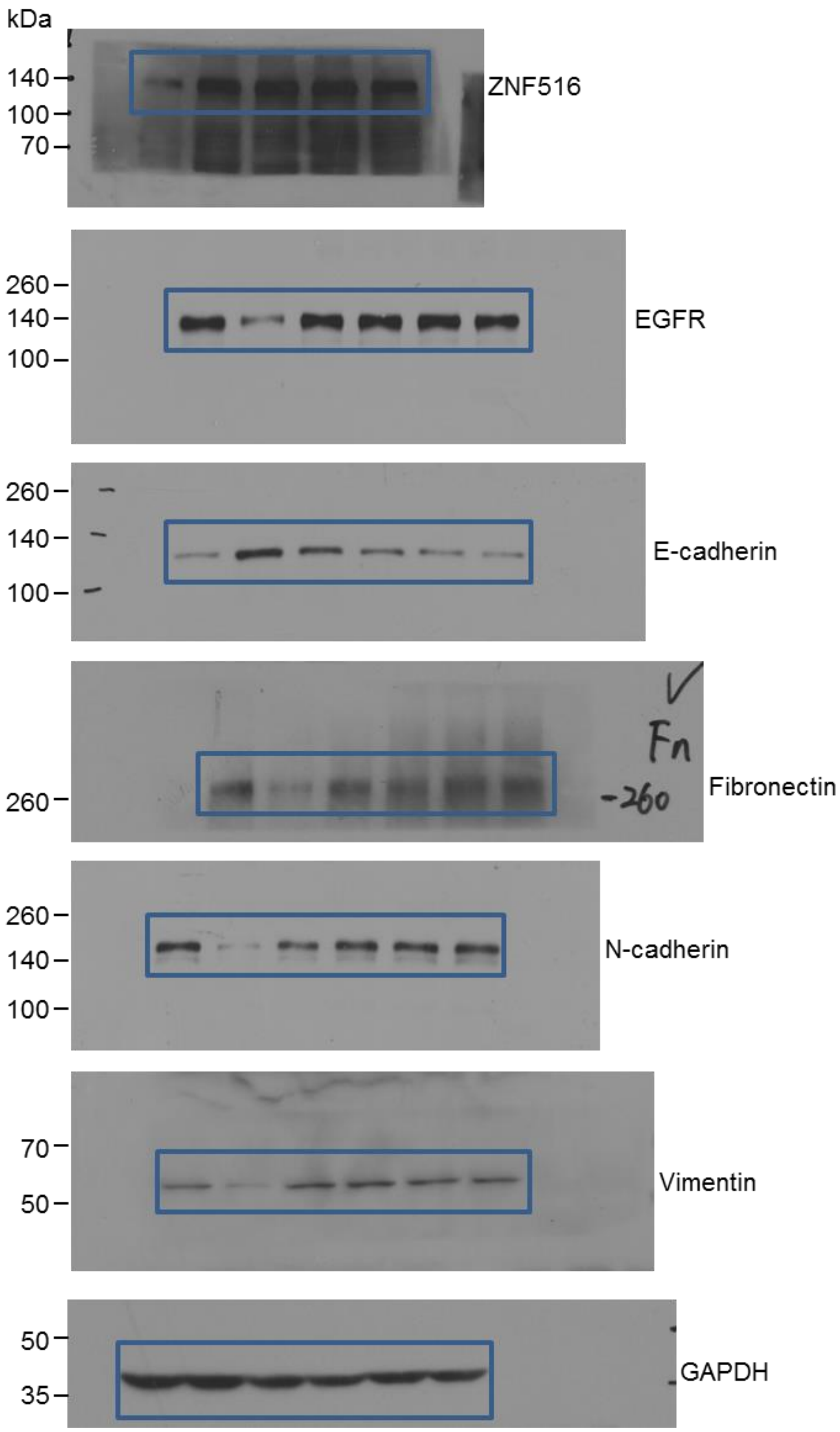

Supplementary Figure 6. Uncropped Scans for Figures 1-3, Figures 5-9, and Supplementary Figures 3-4. The relevant figures are indicated in the blot titles. The cropped areas within the blue boxes are indicated.

	Mean (Relative protein level)		*p* value (T/A)	*r* (Pearson) Correlation with ZNF516	*r* (Spearman) Correlation with ZNF516
	T	A			
ZNF516	0.641	2.882	< 0.001	1	1
EGFR	1.498	0.142	< 0.001	-0.323 (p = 0.037)	-0.482 (p = 0.001)

Supplementary Table 1, related to Figure 9b: Correlation Analysis between ZNF516 and EGFR Expression in Breast Cancer versus Adjacent Normal Breast Tissues. Quantitation was done by densitometry and expressed as signals of ZNF516 or EGFR to GAPDH in the Western blotting analysis, and the correlation between ZNF516 and EGFR was analyzed by Pearson or Spearman correlation.
